# Composite pleomorphic xanthoastrocytoma-epithelioid glioneuronal tumor with *BRAF* V600E mutation – report of three cases 

**DOI:** 10.5414/NP300679

**Published:** 2013-12-10

**Authors:** Dara L. Aisner, Kathy L. Newell, Ania G. Pollack, Bette K. Kleinschmidt-DeMasters, Gary K. Steinberg, Lawrence T. Smyth, Hannes Vogel

**Affiliations:** 1 Departments of Pathology,; 4 Neurology,; 5 Neurosurgery at the Anschutz Medical Campus, University of Colorado at Denver, Aurora, CO, and Departments of; 2 Pathology & Laboratory Medicine, and; 3 Neurosurgery, University of Kansas Medical Center, Kansas City, KS,; 6 Department of Pathology,; 7 Neurosurgery Stanford University, Palo Alto, CA, Department of Pathology, and; 8 Kaiser Permanente Redwood City Medical Center, Redwood City, CA, USA

**Keywords:** pleomorphic xanthoastrocytoma, epithelioid, BRAF V600E, glioneuronal, composite tumor

## Abstract

We report three examples of a composite pleomorphic xanthoastrocytoma-epithelioid glioneuronal tumor (PXA-EGT) occurring in an adolescent male and two young women. All were superficial and two were located in proximity to the optic nerves. Previously reported composite PXA-gangliogliomas (PXA-GG), have been considered “collision tumors” since little intermingling of the two elements has been present. In contrast, we hypothesized that the two elements of the PXA-EGT might instead derive from a common origin. To test this, we sampled the separate regions of these biphasic tumors and assessed each component for the *BRAF* V600E mutation, a genetic feature seen in two-thirds of pure PXAs. The *BRAF* mutation was found in both tumor areas in all cases, suggesting a common origin for the components, rather than a collision tumor. These biphasic PXA-EGT cases represent a new histomorphological combination of neuroepithelial neoplastic elements. These cases further expand the range of glial neoplasia in which epithelioid morphology is encountered, and add to the growing list of biphasic tumors harboring the *BRAF* V600E mutation.

## Introduction 

When epithelioid morphology is encountered by neuropathologists in central nervous system (CNS) neoplasms, diagnostic considerations usually include high-grade neoplasms such as metastatic carcinoma and melanoma, epithelioid glioblastoma (E-GBM) [1, 2, 3, 4, 5, 6, 7], or malignant rhabdoid tumors including the atypical teratoid/rhabdoid tumor (AT/RT) [8, 9, 10] and others [11, 12]. Although epithelial properties of GBMs have been well described [4, 13, 14, 15, 16], epithelioid morphology in GBMs receives little mention in the 2007 World Health Organization classification of central nervous system (CNS) tumors [17]. Rodriguez et al. [4] distinguished histological, immunohistochemical, and genetic features of E-GBMs from adenoid GBMs or true epithelial GBMs, defined as requiring the identification of epithelial differentiation including nests of cells, squamoid differentiation or true glandular structures, plus immunohistochemical expression of one or more specific epithelial markers. A further distinguishing feature is that while epithelial GBMs are often associated with a sarcomatous element [4, 13, 16], this is far less frequent in E-GBMs [4, 6]. 

We observed three remarkably similar biphasic pleomorphic xanthoastrocytoma-epithelioid glioneuronal tumors (PXA-EGT) in young adults of predominantly low-grade histology that do not conform to previously-described high-grade epithelioid glial neoplasms, including epithelioid or rhabdoid GBMs, AT/RT, or recently-described mixed glioneuronal tumors [18]. The tumors were assessed by immunohistochemistry, electron microscopy in one, immunostaining for INI-1, and polymerase chain reaction (PCR) testing for the *BRAF* V600E mutation after microdissection and separate analysis of both the PXA and EGT components in all three cases. We hypothesized that the results would indicate whether these cases represented a collision tumor of disparate histogenesis whereby only the PXA portion would bear the *BRAF* mutation, versus providing support for interpreting these as a newly recognized biphasic type of CNS neoplasm if both portions contained the same mutation. The *BRAF* V600E mutation was tested because of its significant association with PXAs of WHO Grade II (up to 66%), and PXA with anaplastic features (PXA-As), in contrast to a smaller percent of gangliogliomas and extra-cerebellar pilocytic astrocytomas bearing the mutation [19, 20, 21]. Previously reported biphasic tumors with a uniform mutational background [19, 22, 23, 24], added to our rationale for performing this testing in these reported cases. 

## Case presentations 

### Case 1 

**Figure 1.**
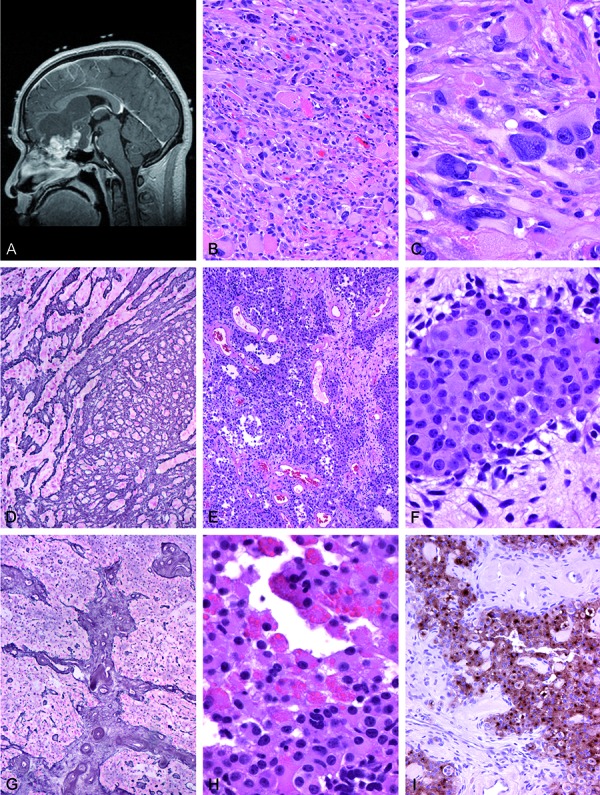
Case 1. A: Sagittal T1-weighted MRI scan with gadolinium enhancement demonstrating a massive cystic and solid, enhancing subfrontal tumor; the cribriform plate was felt to be intact intraoperatively. B: Low-power microscopic view of the pleomorphic xanthoastrocytoma (PXA) portion of the tumor demonstrating numerous eosinophilic granular bodies and a highly cytologically pleomorphic tumor cell population. H&E, 200×. C: High-power photomicrograph of the PXA portion shows the classic features of cytoplasmic xanthic change, as well as occasional nuclear pseudoinclusions. H&E, 600×. D: Reticulin staining of the PXA portion reveals increased reticulin fiber deposition. Gomori reticulin stain, 100×. E: The second, epithelioid portion of the tumor was composed of a monotonous population of smaller epithelioid cells demarcated by hyalinized vasculature; note absence of lymphocytes in this area. H&E, 200×. F: High-power photomicrograph of the epithelioid tumor portion shows rounded cell contours, large nuclei, little or no pleomorphism, and small discrete nucleoli. H&E, 600×. G: This second epithelioid portion of the tumor lacked the rich reticulin network between tumor cells as was present in the PXA areas (compare with Figure 1D), and instead highlighted a vaguely lobular pattern. Gomori reticulin stain, 100×. H: The transition between the PXA areas and the areas with smaller epithelioid cells was narrow, and this zone showed some cells containing numerous small refractile intracytoplasmic eosinophilic granular bodies. H&E, 600×. I: Strong immunoreactivity for synaptophysin was identified in the epithelioid areas of tumor, although mature ganglion cells were not present. Synaptophysin immunostaining with hematoxylin counterstain, 200×.

The patient is an adolescent male who presented in 2009 at the age of 17 years with a recent onset of severe headaches, photophobia, neck stiffness, nausea, and vomiting. Past medical history was significant for an osteochondroma resected from the right scapula earlier that same year. Magnetic resonance imaging (MRI) was performed soon after presentation and showed a lobulated, cystic and solid, enhancing mass with complex features which measured 7.0 × 4.5 × 6.6 cm, centered in the right anterior cranial fossa (Figure 1A). The inferior margin of the mass paralleled the cribriform plate and, by imaging, there was a question of whether the mass extended across the boney skull base and into the right ethmoid sinus. There was compression of the optic chiasm, but the tumor did not appear to be arising from it. 

At surgery, the tumor showed dense adhesion to the optic chiasm and both optic nerves. Only a single (right) olfactory nerve could be identified. A portion of the tumor appeared to be fibrotic and because of the dense adhesion, gross total resection was not attempted. Instead, a minute amount of tumor was left in place, in an attempt to preserve vision. The floor of the anterior cranial fossa was very unusual and irregular in architecture but the surgeon did not feel that the cribriform plate had true boney invasion. The residual tumor tissue has been followed on serial MRIs for 4 years with no evidence of tumor growth. 

Histologically, the tumor showed two very different appearing areas in close juxtaposition, with a clear-cut transition between the two zones and little intermingling of elements. The PXA portion demonstrated pleomorphic cells with lipidized cytoplasm, nuclear pseudoinclusions, reactive lymphocytes and eosinophilic granular bodies (Figure 1B, Figure 1C), and virtually absent mitotic activity. Abundant reticulin was interspersed between cells (Figure 1D). Glial and neuronal differentiation was demonstrated by strong immunoreactivity for glial fibrillary acidic protein (GFAP) and S100 expression, with faint to equivocal focal EMA immunostaining. No overt ganglionic differentiation was seen in the PXA portion of the tumor. Despite this, scattered cells manifested immunoreactivity for synaptophysin, NeuN, and phosphorylated neurofilament protein. 

The second portion of the tumor, in contrast, was composed of a monotonous population of smaller epithelioid cells demarcated by hyalinized vasculature (Figure 1E). Lymphocytes were not seen in this area. The epithelioid tumor cells showed rounded cytoplasmic contours, large nuclei, little or no pleomorphism, and discrete nucleoli (Figure 1F). Although this second portion lacked the rich reticulin network between the tumor cells present in the PXA areas, reticulin staining highlighted the vaguely lobular pattern (Figure 1G). No necrosis, microvascular proliferation, or mitotic activity was present. Eosinophilic fibrillary neuropil, larger neoplastic ganglion cells, microcystic change, and calcifications were entirely absent. 

**Figure 2.**
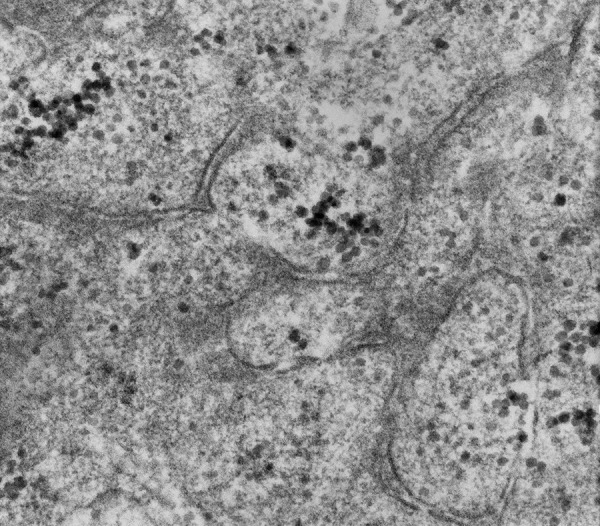
Numerous dense core vesicles are seen within tumor cell processes from the epithelioid portion of the tumor, corroborating synaptophysin immunostaining (see Figure 1I) and verifying neuronal lineage in this portion of the tumor. Direct magnification, 61,500×.

Only a narrow transition zone could be found between the PXA areas and the areas with smaller epithelioid cells. This zone manifested as an admixture of multinucleated and pleomorphic cells and showed some cells containing numerous small refractile intracytoplasmic eosinophilic granular bodies (Figure 2H). Also noted in the transition zone were scattered cells with vacuolization and xanthic change. Large neoplastic ganglion cells and fibrillary neuropil were not identified in the transition zone. 

The small epithelioid areas of tumor had strong diffuse immunoreactivity for S100, CD56, and synaptophysin (Figure 1I), with lesser GFAP immunostaining, estimated at 1/5 of the small epithelioid cells. Unlike the PXA portion of the tumor, NFP and NeuN were not identified in the epithelioid cells. CD34 highlighted the small vasculature in the hyalinized septae in the epithelioid areas, and was strongly positive in a thin hypercellular layer of stromal cells at the edges of the septae; these cells were interpreted as parts of the vascular septae since they lacked mitotic activity or MIB-1 labeling, as would have been expected with stem-like cells. MyoD1, desmin, and smooth muscle actin were negative in all tumor cells in both areas. MIB-1 was 1% in both portions of the tumor, and nuclear INI-1 protein was retained in all tumor cells in both areas. 

Electron microscopy demonstrated numerous intracytoplasmic neurosecretory granules, verifying neuronal lineage in the epithelioid cells of this low-grade glioneuronal tumor (Figure 2). 

Because the tumor possessed two geographically distinct areas of PXA versus epithelioid neuronal tumor, samples could be tested separately and confidently from these two differing areas of tumor for *BRAF* V600E mutational status. Thus, two separate tumor DNA samples from each area were obtained after microdissection using paraffin sections deparaffinized in xylene, hydrated through graded alcohols to water, and stained with Gill’s hematoxylin. 

**Figure 3.**
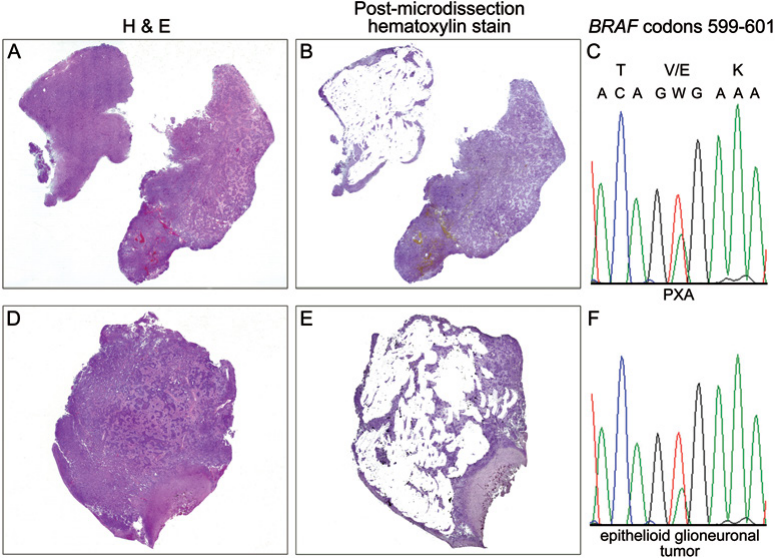
*BRAF* c.1799T>A (p.V600E) mutation is present in areas with both “typical” PXA morphology and epithelioid glioneuronal components. A, D: Scanning power of two different areas of tumor present in different blocks from the same case with A) having the “typical” PXA morphology and D) demonstrating epithelioid morphology (see Figure 1 for higher-power images of these areas). B, E: Scanning power of the same regions shown after microdissection was performed to demonstrate the relatively high fidelity of the microdissection. C, F: Electropherograms demonstrating the presence of a T > A mutation at position c.1799 of *BRAF*, resulting in an anticipated p.V600E mutation.

For direct sequencing, template DNA was PCR amplified using forward (5’TGCTTGCTCTGATAGGAAAAT3’) and reverse (5’TCAGGGCCAAAAATTTAATCA3’) *BRAF* exon 15 primers. The *BRAF* c.1799T>A (p.V600E) mutation was identified in both the PXA and EGT areas of the case (Figure 3). 

#### Case 2 

**Figure 4.**
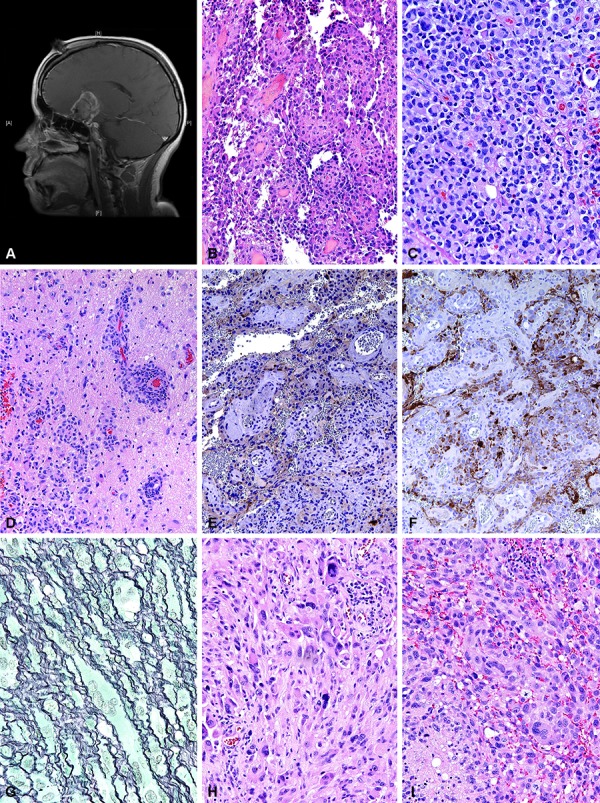
Case 2. A – F: Primary tumor; G – I: Recurrence. A: Parasagittal T1-weighted MRI scan with gadolinium enhancement demonstrating a cystic and solid, enhancing subfrontal tumor, found intraoperatively to involve the frontal lobe and optic nerves. B: Low-power microscopic view of the original resection showing papillary growth and epithelioid cells. H&E, 200×. C: Higher-power view of epithelioid morphology, with vague fibrovascular septae enclosing sheets of monotonous cells, some with nuclear displacement by eosinophilic cytoplasm. H&E 200×. D: Infiltrative growth of the epithelioid cells. H&E, 100×. E: Synaptophysin immunostaining showed weak but distinct immunostaining of the majority of cells within the regions of papillary growth. 100×. F: GFAP immunostaining identifies a subset of strongly expressing cells in the same region depicted in D, 100×. G: Recurrent tumor. Higher-power photomicrograph showing typical features of a PXA, with marked nuclear pleomorphism, intranuclear inclusions, and EGBs. H&E, 200×. H: Higher-power view of pleomorphic cells associated with pericellular reticulin deposition. Gomori reticulin stain, 400×. I: Anaplastic features were noted in the recurrence, with necrosis and increased mitotic activity. H&E, 200×.

The patient was a 24-year-old woman who developed headache accompanied by language impairment and right hemiparesis. A CT scan revealed a left basal ganglia hemorrhage. An MRI angiogram revealed what was thought to represent unilateral left-sided moyamoya disease. Two months later, a microscopic extracranial to intracranial revascularization and anastomosis procedure was performed with improvement in symptoms. Four to 5 months later she had an increase in her headaches. An MRI revealed a heterogeneous left frontal lobe mass with hemorrhage, calcification, mass effect, and significant surrounding edema. Upon re-review of her original imaging, it was apparent that she had a left mesial frontal tumor which had been previously obscured by hemorrhage. The tumor exhibited significant interval growth with edema. A left frontotemporal craniotomy with partial resection was performed. Pathological analysis revealed a focally papillary tumor with epithelioid cells lining the papillae (Figure 4B) as well as filling intervening areas (Figure 4C). Perivascular invasion of adjacent brain was noted. (Figure 4D). No significant pleomorphism or other features conventionally associated with PXA were noted with complete sampling of the specimen. There was variable immunopositivity for synaptophysin (Figure 4E) as well as focal GFAP staining in a subset of cells (Figure 4F). The tumor was diagnosed as a low-grade glioma, favoring a papillary glioneuronal tumor. 

Eight months later, her headaches worsened and an MRI showed a slight interval increase in the size of her tumor (Figure 4A). The following month, the patient underwent left frontotemporal craniotomy. Intraoperatively, a 4.5 cm, firm, vascular tumor was found in the left mesial anterior basal frontal lobe. The tumor had invaded the walls of the left supraclinoid internal artery, left M1 middle cerebral artery, and left A1 anterior cerebral artery, and it infiltrated and expanded the left optic nerve yielding the intraoperative impression that the tumor arose from the left optic nerve itself. The tumor was densely adherent to the surrounding dura overlying the planum sphenoidale. The tumor extended to the frontal horn of the left lateral ventricle, causing significant mass effect on the surrounding frontal lobe. 

Pathological analysis showed a significant difference in microscopic appearance, with areas of marked nuclear pleomorphism (Figure 4G), some lipidized cells, and eosinophilic granular bodies with the epithelioid morphology noted in the prior specimen greatly underrepresented but present focally. Pericellular reticulin deposition was focally very prominent (Figure 4H). In addition, scattered mitotic figures, up to 14 per 10 high power field, and focal necrosis without microvascular proliferation (Figure 4I) were noted and the diagnosis was revised to PXA with focal anaplastic features. The EGT morphology which dominated the initial resection was only present in focal regions that were distinct and not intermingled with the PXA portions. 

BRAF V600E mutational analysis was performed separately on both the original and subsequent specimens, using areas exclusively representative of the glioneuronal and PXA morphologies. Detection of the BRAF V600E (1799T>A) mutation was performed using a modified version of published allele-specific PCR methodology [25]. 

#### Case 3 

**Figure 5.**
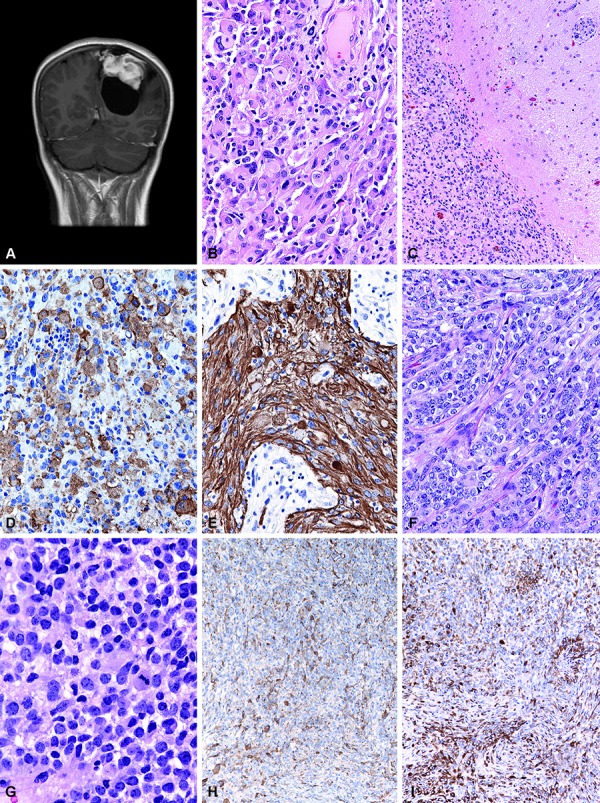
Case 3. A – D: Primary tumor: E – I: Recurrence. A: Coronal T1-weighted MRI scan with gadolinium enhancement of a cystic mass with enhancing solid portion arising in the left parieto-occipital region, abutting the inner table of the parietal skull and superior sagittal sinus. B: Photomicrograph of the primary tumor showing typical features of a PXA. H&E, 200×. C: Subarachnoid spread of PXA. H&E, 100×. D: Synaptophysin. E: GFAP immunostain, 200×. F: Recurrent tumor showed sheets of epithelioid cells in vague nests divided by fibrovascular septae. H&E, 200×. G: The recurrent tumor showed areas of mitotic activity up to 3/10 HPF. 200×. H: Synaptophysin. I: GFAP immunopositivity in the epithelioid recurrent tumor, 100×.

A 22-year-old woman was found to have a left parieto-occipital tumor upon investigation of an acute change of mental status, speech arrest, and right sided hemiparesis affecting her arm more than her leg. She also had a long history of migraine headaches that had recently intensified. The tumor was characterized as a 7.4 × 4.4 × 7.2 cm mass with a large cystic component and contrast-enhancing solid portion. The mass abutted the inner table of the parietal skull and superior sagittal sinus (Figure 5A). A gross total resection was performed, with close observation postoperatively and no radiation therapy. Histologically, the tumor demonstrated marked pleomorphism (Figure 5B), lipidized cells, eosinophilic granular bodies, and subarachnoid spread (Figure 5C). Focal synaptophysin (Figure 5D) and fascicles of GFAP-immunopositive tumor (Figure 5E) were noted. The tumor was diagnosed as a PXA. Approximately 3 years later, MRI studies showed a small area of contrast enhancement in the bed of the previous resection, which underwent surgical resection. This specimen did not display any features of a PXA. Rather, it showed sheets and fascicles of epithelioid cells without significant pleomorphism (Figure 5F), focally prominent mitotic activity up to 3 mitoses per 10 high power fields (Figure 5G), with focal synaptophysin (Figure 5H), and GFAP (Figure 5I) immunopositivity. 

Separate samples from the primary and recurrent tumors were tested by extracting genomic DNA from slides of formalin-fixed, paraffin-embedded tumor tissue and analyzed by multiplex PCR amplification and a single nucleotide primer extension assay followed by capillary electrophoresis for signal detection of the presence or absence of the *BRAF* V600E mutation. The primary and recurrent tumors were both positive for the mutation. 

## Discussion 

We report three examples of a unique brain tumor occurring in young adults. Two developed in the optic nerve region and the third was parieto-occipital but all three showed the characteristic superficial location and subarachnoid spread of conventional PXA. The histology appears to be unique and unreported, and is characterized by composite juxtaposition of classic PXA areas adjacent to epithelioid glioneuronal tumor, or in the two examples with recurrence, almost exclusively PXA morphology in one occurrence and EGT in the other. The PXA component of Case 2 which accounted for the predominant histology in the recurrence achieved the published criteria for PXA with anaplastic features [26]. Case 3 adhered to the usual tendency of some PXAs to assume a more monotonous appearance with closely packed cells and increased mitotic activity and necrosis approaching anaplasia which nonetheless did not reach the 5 mitoses per 10 high power fields required for designation of anaplastic features [26]. Yet it retained a distinctive glioneuronal immunohistochemical profile not classically associated with anaplastic transformation. 

These cases add to the known propensity for the PXA to have composite elements, principally ganglioglioma [27], but also with an oligodendroglial element [28], highly vascularized “angiomatous” forms [29], and a composite tumor with combined dysembryoplastic neuroepithelial tumor (DNT) and PXA [30]. We have not identified previous reports of PXA-GGs that mention epithelioid morphology in either the PXA or the GG portion of the tumor. Similarly, epithelioid morphology in pure PXA is recognized but an unusual feature. In 1991, Kepes [31] makes passing reference to his own material which included a case of PXA with “epithelioid” features and cites the report by Iwaki et al., “where the otherwise typical tumor cells of PXA are more clustered by connective tissue septa and in the process assume a more “epithelioid character…” [32]. Review of the publication by Iwaki et al. [4] shows cases with similar fibrovascular septae to our current cases, but what the authors themselves considered to be epithelial rather than epithelioid morphology, notwithstanding the lack of evidence justifying the formal designation of epithelial in a glial neoplasm. Furthermore, previous descriptions of epithelioid morphology in PXAs do not specify glioneuronal differentiation defined in these three cases by synaptophysin immunohistochemistry, notwithstanding the fact that synpatophysin immunopositivity does not always strictly equate with neuronal differentiation, such as in the examples of pilomyxoid astrocytomas [33] and choroid plexus papillomas [34]. We also do not feel that these PXA-EGTs conform to other newly-described mixed glioneuronal tumors [18], both on histological and molecular genetic grounds. 

Our studies to test each component for a common genetic origin indicated that both the PXA and EGT components in all of the tumors were positive for the *BRAF* V600E mutation. It should be emphasized that this mutation is not invariably present in all PXAs [19, 20, 21]. Schindler et al. [21] investigated a large number of PXAs (64: 38 adult, 26 pediatric) and PXA-As (23: 13 adult; 10 pediatric) and found the mutation in 66% of PXAs overall (63% adult, 69% pediatric) and 65% of PXA-As (38% adult, 100% pediatric). Thus the mutation could be found equally in low- (Grade II) and higher-grade (paralleling WHO Grade III) PXAs. In contrast, Dias-Santagata et al. [20] found the mutation in 60% of WHO grade II PXAs, but only in 17% of PXA-As. Differences may relate to the fact that only 6 PXA-As were available for analyses in the latter study. Dougherty et al. found [19] the same *BRAF* V600E mutation in 9 of 18 pure pediatric GGs in their larger tumor cohort, none of which had a more adverse clinical outcome compared to non-mutated pediatric GGs, although the 3-year-follow-up time was short and not complete for the entire group. We are not aware of published reports of a similar analysis of PXA-GG in which both components were tested separately for the *BRAF* V600E mutation, but would propose this investigation as an interesting comparison to the findings in these cases. 

An interesting aspect of *BRAF* V600E is the fact that several biphasic tumors have been reported with this mutation, suggesting that perhaps this mutational underpinning allows for emergence of additional tumor components. It must be emphasized, however, that previous reports have not been a low-grade tumor (PXA or GG) co-associated with another similar low-grade tumor, but rather with a malignant AT/RT co-associated with PXA or GG. The first report to describe progression of a GG to AT/RT was published in 2006 [22]. In that patient, the original tumor had been diagnosed as an optic pathway glioma but in retrospect was felt to be an under-sampled GG. After multiple chemotherapy regimens and radiation therapy, the tumor recurred 9 years later and was found to be a mixed GG-AT/RT. In 2011, we reported a mixed GG-AT/RT in which both components were identified at clinical onset [23]. A case of a PXA-AT/RT was reported by Chacko et al. in 2007 [24]. Using FISH techniques, they detected monosomy 22 in most of the rhabdoid cells and disomy in 50% of the cells of the glial component, with varying gains of chromosome 22 in the remainder of the PXA cells. They felt that the AT/RT portion arose from the PXA, likely the diploid cells rather than the cells with the multiple copies of chromosome 22. An additional case of mixed PXA-AT/RT reported by Dougherty et al. [19] was investigated for the *BRAF* V600E mutation, as well as the two previously reported cases of Allen et al. [18] and Chacko et al. [24]. Both of these previously published cases, plus their new additional case mentioned in their report (n = 3), were found to contain the mutation. Thus, to date, two GG-AT/RTs and two PXA-AT/RTs have been investigated, all with *BRAF* V600E mutation and additional loss of INI-1 in the AT/RT component. These observations, when added to the findings in our cases, underscore the propensity of some CNS tumors with the *BRAF* V600E mutation to display composite or divergent morphological components. They also demonstrate epithelioid morphology that is not associated with high grade, and illustrates a unique neuronal morphology associated with epithelioid differentiation in PXA. 

## Acknowledgments 

The authors gratefully acknowledge Ms. Lisa Litzenberger for photographic expertise, Mrs. Diane Hutchinson for manuscript preparation, and Norman Cyr for expert assistance in figure preparation. 

## Conflict of interest 

The authors declare no conflict of interest.
